# A Novel Protocol for Integrated Assessment of Upper Limbs Using the Optoelectronic Motion Analysis System: Validation and Usability in Healthy People

**DOI:** 10.3390/bioengineering12090905

**Published:** 2025-08-23

**Authors:** Luca Emanuele Molteni, Luigi Piccinini, Daniele Panzeri, Ettore Micheletti, Giuseppe Andreoni

**Affiliations:** 1 Scientific Institute IRCCS “E.Medea”, Bosisio Parini, 23842 Lecco, Italy; luigi.piccinini@lanostrafamiglia.it (L.P.); giuseppe.andreoni@polimi.it (G.A.); 2 Department of Design, Politecnico di Milano, 20133 Milano, Italy

**Keywords:** upper limb, functional assessment, kinematic analysis, shoulder kinematics, elbow kinematics, wrist kinematics

## Abstract

(1) Background: Upper limb (UL) function plays a central role in daily life, enabling essential tasks such as reaching, grasping, and eating. While numerous tools exist to evaluate UL kinematics, their application in pediatric populations is often limited by a lack of age-specific validation. This study presents a novel motion analysis protocol featuring a customized marker set, aimed at assessing UL movements in the three anatomical planes across different age groups, with a focus on pediatric applicability. (2) Materials and Methods: A SmartDX motion capture system was used, with 30 markers positioned on the upper body, referencing the trunk as the root of the kinematic chain. Ten healthy participants (mean age: 18.69 ± 12.45 years; range: 8.0–41.4) without UL impairments were recruited. The broad age range was intentionally selected to assess the protocol’s transversal applicability. (3) Results: Results showed excellent intra-operator reliability for shoulder and wrist kinematics (ICC > 0.906) and good reliability for elbow movements (ICC > 0.755). Inter-operator reliability was good to excellent (shoulder ICC > 0.958; elbow ICC > 0.762; wrist ICC > 0.826) Usability, measured via the System Usability Scale, was rated as good (83.25). (4) Conclusions: The proposed protocol demonstrated strong reliability and practical usability, supporting its adoption in clinical and research settings. Its design allows for adaptability across motion capture platforms, promoting wider implementation in pediatric UL functional assessment.

## 1. Introduction

Upper limb (UL) function is essential for performing everyday tasks such as pointing, grasping, handling, and eating. Accurate assessment of these movements is crucial in clinical medicine, rehabilitation, and ergonomics, particularly for tailoring therapeutic interventions in conditions like stroke and cerebral palsy (CP) [1,2]. Studies estimate that 50–85% of individuals with acute stroke and around 50% of those with chronic stroke exhibit UL impairments, which significantly affect independence and quality of life [3,4,5].

Various clinical tools are available to evaluate UL function [6], and guidelines emphasize the importance of using valid, reliable, and responsive outcome measures [7,8]. The literature proposes different methods like: The Melbourne Assessment of Unilateral Upper Limb Function (MUUL), Quality of Upper Extremity Skills Test (QUEST), and Shriners Hospital Upper Extremity Evaluation (SHUEE) [9,10,11]. These methods qualitatively assess motor performance of the upper extremity during different functional tasks [8]. However, these methods provide only general results, as they rely on weighted scores based on evaluator experience [12]. Distinguishing motor recovery from compensation can be difficult for these measures, which is crucial in therapies such as constraint-induced movement therapy (CIMT), trunk restraint therapy (TRT) and bilateral arm therapy (BAT) [5,13].

Combining clinical outcome measures with kinematic analysis is increasingly recognized as a valuable tool for assessing UL movement, with growing support from expert consensus and clinical research [14]. By providing objective, quantitative data on motor performance, kinematic analysis allows clinicians and researchers to better characterize recovery patterns, identify compensatory mechanisms, and improve prognostic accuracy. Kinematic assessments describe the biomechanical properties of movement with precision, offering accurate and reproducible information on motor control and coordination [15]. These metrics are critical for understanding dysfunction in specific anatomical regions and contribute to informed clinical decision-making regarding surgical intervention, pharmacological treatment, or rehabilitation planning [16]. For example, kinematic motion analysis is widely applied in conditions such as cerebral palsy (CP) to monitor motor performance during daily activities, support individualized rehabilitation strategies, and objectively evaluate treatment outcomes [17].

Kinematic metrics such as speed, smoothness, efficiency, accuracy, control strategy, and functional range of motion are often used in combination with clinical scales to evaluate UL function in patients with neurological conditions [18]. These tools allow for the assessment of reaching, gross, and fine motor abilities, as well as spatiotemporal movement characteristics. Jaspers et al. highlighted the importance of integrating kinematic data with qualitative scales to obtain a comprehensive picture of upper extremity function, which is essential for selecting the most appropriate treatment approach in pediatric patients with CP [17].

Despite these advantages, evidence regarding the responsiveness of kinematic measures in UL assessment remains limited [19]. Recent recommendations encourage standardization of kinematic analysis measurement protocols according to International Classification of Functioning (ICF) levels, tasks and conditions analyzed as well as psychometric properties [14,20]. Optoelectronic systems are considered the gold standard for UL kinematic analysis, offering high accuracy and temporal resolution [2]. However, standardized and validated protocols remain scarce. Implementing a standardized protocol to measure UL movement across three anatomical planes and to monitor the spatiotemporal characteristics of movement could serve as a valuable clinical tool. Combined with clinical scales, it can support the personalization and ongoing monitoring of rehabilitative interventions.

The aim of this study is to introduce and validate a novel motion analysis protocol for the UL, based on a dedicated marker set and an integrated biomechanical model. This protocol is designed for the global assessment of UL kinematics during functional movements, such as reach-to-target tasks, enabling a more detailed and objective evaluation of motor behavior and its potential abnormalities. Such detailed characterization is essential for improving the understanding of UL functionality in both healthy and clinical populations. Before clinical implementation, a newly developed protocol must undergo rigorous validation. Specifically, its repeatability—both intra- and inter-operator—must be established through testing on healthy participants. This study describes the methodological development of the protocol and presents the results of its validation in terms of accuracy, repeatability, and usability, considering both the experience of the operators and the feasibility for the subjects.

## 2. Materials and Methods

### 2.1. Subjects

To validate the protocol in both adults and children, ten healthy subjects (5 males, 5 females; mean age: 18.69, SD: 12.45; age range: 8.0–41.4) without any UL functional limitations voluntarily participated in the study. Informed consent was obtained from all adult participants and from the parents or legal guardians of the minors.

Inclusion criteria included the absence of functional impairments in the ULs. Exclusion criteria included behavioral, visual, or auditory disorders, as well as the presence of pain during UL movements. Baseline characteristics of the sample are presented in Table 1.

The study was approved by the Ethics Committee of the Institute (Approval Number: 55/23, Comitato Etico dell’Associazione “La Nostra Famiglia”—IRCCS “Eugenio Medea,” 18 May 2023). The clinical trial has been registered on Clinical Trials.gov: NCT06400667.

### 2.2. Data Collections

Various protocols for UL kinematic assessment have been proposed in the literature; however, they often evaluate motion only in the sagittal plane and typically exclude trunk and head kinematics [21,22]. The present study aimed to develop a general-purpose protocol capable of capturing UL movement in all three anatomical planes—sagittal, frontal, and transverse—for the humerus, forearm, and hand. Additionally, trunk and head kinematics were included to capture the core contribution to motor behavior.

**Figure 1:**
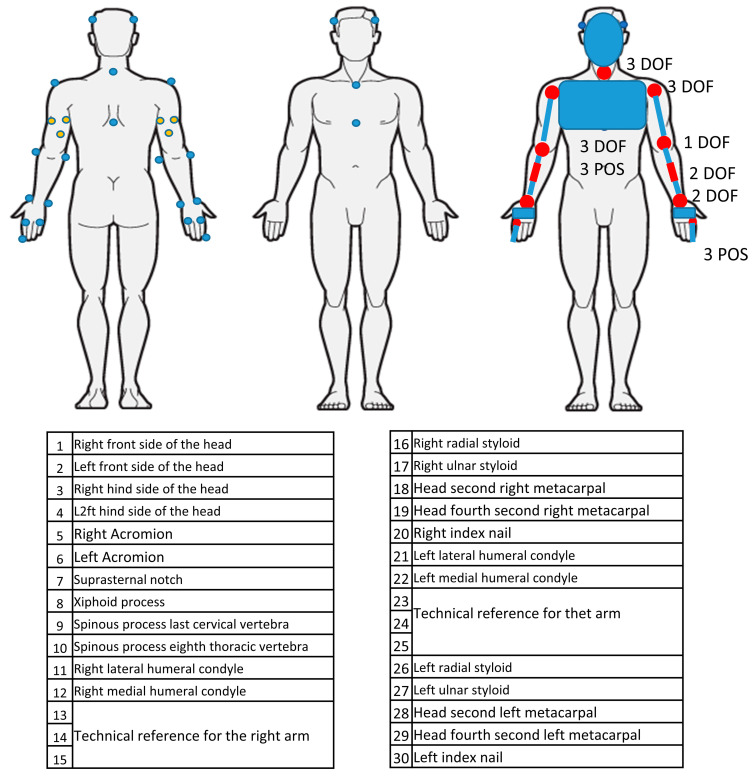
Positions of the markers on the subject and corresponding biomechanical model.

The protocol was designed to be applicable across the lifespan, including pediatric subjects. Therefore, special attention was paid to marker size, visibility, and placement to minimize occlusion and overlap. The anatomical coordinate system of each body segment was calculated by placing 28 reflective markers (10 mm diameter hemispherical spheres) [23]. Marker placement and the resulting biomechanical model are illustrated in Figure 1.

The model consists of 8 segments and allows the computation of 9 positional degrees of freedom (trunk position and fingertip position) and 22 joint angles. Segment reference frames were defined for the humerus, forearm, hand, thorax, and head following International Society of Biomechanics (ISB) guidelines [23,24]. Each segment, considering the relevant anatomical reference system, was calculated, as shown in Table 2. In particular, for the humerus, two distinct reference systems were used to calculate shoulder and elbow angles, respectively [25].

Obliquity, flexion-extension, and rotation angles of each segment were computed relative to adjacent segments or a global reference system, using Euler angles in the order: sagittal (flexion/extension), frontal (lateral bending), and transverse (axial rotation), following ISB recommendations [23].

**Figure 2:**
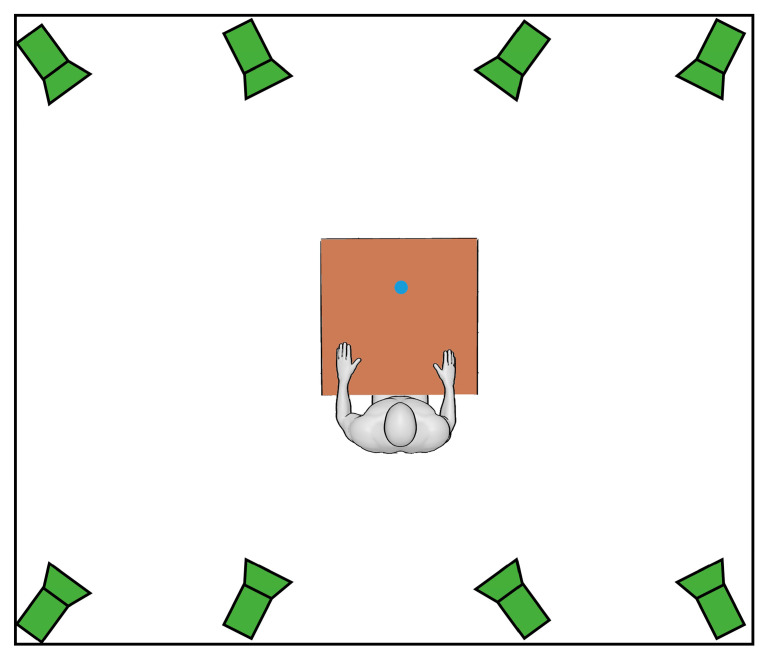
Laboratory setup and camera positions.

Motion data were captured using an optoelectronic motion capture system (SMART DX, BTS Bioengineering, Milan, Italy) equipped with eight infrared cameras operating at 60 Hz. The cameras were positioned on the front and side walls of the room at approximately 2 m from the subject (Figure 2). Markers were placed directly on the subject’s skin by trained physiotherapists experienced in anatomical landmark identification and motion analysis procedures.

During acquisition, subjects were seated on a stool with their forearms resting on a table adjusted to navel height. During the test, the subject is required to reach a target on the table using the index finger. This ensures that all joints of the UL are engaged and that the movement is standardized. The target was placed at 80% of the subject’s arm’s length, over a cylinder with a height of about 8 cm, placed on the table. Each subject completed five “Hit-to-Target” (HtT) trials with the right arm, followed by five trials with the left arm. Each session was repeated twice by two different operators, resulting in a total of 20 trials per arm per subject.

The development of a new protocol of analysis requires the validation of its repeatability before its definitive and clinical application. The repeatability of the protocol was assessed by analyzing variability in outcome measures across sessions and between operators [26]. Specifically, both intra-operator (same operator, repeated sessions) and inter-operator (different operators) repeatability were evaluated. The experimental protocol for the intra- and inter-operator validation consisted of the following steps:

1. Preparation of the subject, positioning the markers (30) on the selected body landmarks.
2. The acquisition of 5 HtT trials with each arm;
3. Detachment of the markers from the subject.

For the analysis of intra and inter operative repeatability, the various steps were repeated 2 times by the same therapist, and 2 times by a different therapist. In total, each subject attended 4 sessions, so we analyzed 20 HtT trials for each arm in each subject. The repositioning of markers by both the same operator and the second operator was carried out in consecutive sessions, with about a three-minute break between each one. The total test time for this repeatability validation was about one hour and 30 min per session.

Usability evaluation is crucial for the successful clinical implementation of medical products [27]. According to ISO 9241–11 [28], usability encompasses effectiveness, efficiency, and user satisfaction. After each acquisition session, the participants were given the System Usability Scale (SUS) [29] 36 and an assessment to evaluate how challenging the different phases of the test were using a 5-point Likert-scale methodology (ranging from 1 to 5). The considered operations were as follows:

- Phase 1: instrument preparation and marker preparation (for the test subjects, the part of instrument preparation was excluded from the questionnaire because the subjects were not involved in this operation);
- Phase 2: marker positioning;
- Phase 3: recording the movements with the optoelectronic system;
- Phase 4: removing the markers.

Each phase was timed to assess the duration required.

### 2.3. Data Processing

Raw data were processed with Smart Analyzer software 1.10.0470 (BTS Bioengineering, Milano, Italy). First, the 3D data were filtered and eventually interpolated in cases of short periods of missing data. Subsequently, movement phases were segmented according to established criteria [30,31]:

- Going Phase: Begins when the velocity of the finger marker exceeds 50 mm/s and ends at the onset of the Adjusting Phase.
- Adjusting Phase: This phase was not defined by velocity thresholds, due to the fluctuating nature of speed during fine adjustments. Instead, it was identified based on the distance between the finger and the target, using a threshold set as the average distance plus three standard deviations across five movements. The end of this phase and the start of the Returning Phase were defined using the same criteria.
- Returning Phase: Starts at the conclusion of the Adjusting Phase and ends when the finger marker speed drops below 50 mm/s.

For each body segment, joint angle was normalized over the movement cycle. Range of motion (ROM) was calculated as the difference between the maximum and minimum angles across the entire movement. Mean angular velocity was computed for each joint and anatomical plane and segmented by movement phase. Additionally, we used other metrics from the literature, as follows:

- End-point (finger) metrics: Derived from finger kinematic data, these metrics assess movement speed, accuracy, efficiency, and smoothness.
- Trunk compensation metrics: Calculated from the marker placed on the sternum, these metrics quantified compensatory trunk movements during reaching tasks.

The metrics used are summarized in Table 3.

### 2.4. Statistical Analysis

For the technical and clinical validation phase, the protocol was tested on a sample of at least 10 participants and two clinical operators, with each procedure repeated five times per condition. This sample size is considered adequate to assess both the technical aspects of the protocol—such as intra- and inter-operator repeatability and measurement variability—and its procedural feasibility in a clinical setting, as reported in previous studies [26,34,35]. Intra- and inter-operator repeatability were evaluated by calculating the correlation coefficients of the average ROM across the five trials within corresponding sessions.

Usability was assessed through descriptive statistics (mean and standard deviation) for perceived usability and task difficulty, along with the average score obtained from the SUS questionnaire [36]. All statistical analyses were performed using SPSS 21 software (IBM Corp., Armonk, NY, USA).

## 3. Results

**Figure 3:**
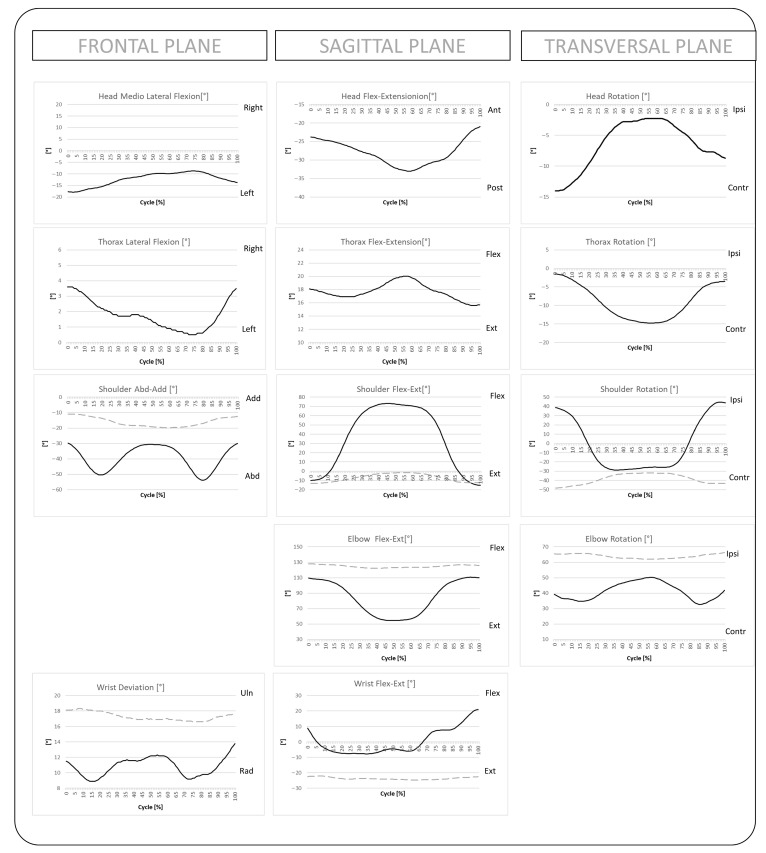
Example of UL angles in all three anatomical planes, normalized on the movement cycle. The black line represents the right UL; the gray dashed line represents the left UL.

The proposed protocol is designed to quantify UL joint angles normalized over the movement cycle during a HtT task. Additionally, this study aimed to validate the kinematics of the UL and related spatiotemporal parameters. An example of the data output and automatically generated clinical report is shown in Figure 3.

To assess repeatability—both intra- and inter-operator—we computed the intraclass correlation coefficient (ICC) using a two-way mixed-effects model with absolute agreement and average measures. ICC values were calculated for the range of motion (ROM) of the UL joints and the extracted spatiotemporal parameters. The interpretation of ICC values followed established guidelines—values between 0.50 and 0.75 indicate moderate reliability, between 0.75 and 0.90 indicate good reliability, and values greater than 0.90 indicate excellent reliability [37].

### 3.1. Time Phases and End-Point Metrics

Regarding the repeatability analysis for intra-operator marking, Table 3 shows the results obtained. All the end-point indexes showed an ICC > 0.867, indicating good to excellent reliability. Only the Number of Velocity Peaks (NVP) parameters showed a lower ICC value of 0.739, indicating moderate reliability, although this value was close to the cutoff limit of 0.75. The time phases showed slightly worse results: reliability was moderate for the total time and for the adjustment phase (ICC > 0.785), while for the other phases, the reliability was also moderate (ICC > 0.725). Even in these cases, however, the ICC values were close to the cutoff limit of 0.75. Regarding the repeatability analysis for inter-operator reliability, the results are very close to the intra-operator reliability. Table 4 presents the obtained results. Similarly, the end-point metrics demonstrated good reliability (ICC > 0.793), except for the NVP, which showed moderate reliability (ICC = 0.721). Similarly to the intra-operator analysis, the time phases showed slightly worse results with moderate reliability.

### 3.2. Trunk Compensation Metrics

Regarding the intra-operator repeatability analysis for trunk compensation indices, Table 5 presents the results obtained for each movement phase. All indices demonstrated good to excellent reliability, with ICC values exceeding 0.79 and reaching a maximum of 0.956. In the inter-operator repeatability analysis, results were slightly lower but still acceptable, with trunk compensation indices showing moderate to good reliability (ICC > 0.696).

### 3.3. Range of Motion

The repeatability analysis for the ROM showed consistent results between intra- and inter-operator assessments. Both the right and left ULs exhibited excellent reliability at the shoulder and wrist joints. Specifically, intra-operator reliability for these segments was ICC > 0.906, while inter-operator reliability was ICC > 0.941. The elbow joint demonstrated slightly lower reliability, showing good intra-operator repeatability and moderate to good inter-operator agreement. Reliability values for thorax and head segments ranged from moderate to good. Table 6 shows the results of ROM for different body segments.

### 3.4. Usability Assessment

To evaluate the practical applicability of the proposed protocol in clinical settings, both operator and patient experiences were assessed. Perceived difficulty during the different phases of acquisition was rated by participants and clinicians, as summarized in Table 7.

The phase1 correspond to the instrument preparation, phase 2 correspond to the subject preparation, phase 3 correspond to recording the movements with the optoelectronic system and the phase 4 correspond to the removing the markers.

The SUS was administered at the end of each session, resulting in an average score of 83.25, indicating a high level of usability. Furthermore, Table 8 presents the average time required for each procedural step, as well as the total duration of the entire acquisition process.

## 4. Discussion

The method presented in this study is a non-invasive and integrated approach for evaluating UL movement during functional tasks such as HtT movement. Its implementation requires a human motion analysis (MA) system, and its relative simplicity—developed in close collaboration with clinicians—makes it suitable for routine use in clinical practice. The proper balance between the need for a detailed motion description (requiring a high number of markers) and the limitation of the complexity (i.e., the number of markers to be prepared and placed onto the subject) not to affect the usability and acceptance in operators and subjects is respected. Indeed, the time needed for the participant for the examination is about 20 min for the entire process (Table 7). The relatively short completion time observed in this study was likely influenced by the characteristics of the recruited participants, who were all healthy and highly cooperative. In clinical populations, particularly in patients with neurological disorders or behavioral impairments, the time required for protocol execution may reasonably be longer. Furthermore, both clinicians and patients perceived the protocol as easy to perform. The usability evaluation, as measured by the SUS, yielded an average score of 83.25, indicating a high level of usability [38]. These results are particularly relevant for pediatric populations, where test duration and simplicity are critical due to reduced cooperation and limited attention spans in children. Short, engaging protocols like this one are likely to improve compliance and measurement accuracy in such groups. No major barriers were identified in this healthy adult cohort. Future clinical populations will be investigated to determine potential challenges, such as attention span, cooperation, or task comprehension, and any differences in perceived usability between children and adults.

In the intra- and inter-operator repeatability analyses, all endpoint indices demonstrated good to excellent reliability (ICC > 0.793), except for NVP, which showed moderate reliability. Trunk compensation indices also demonstrated good to excellent intra-operator reliability and moderate to good inter-operator reliability. Only two specific metrics—the medio-lateral displacement during the Going Phase and the antero-posterior displacement during the Adjusting Phase—showed ICC values close to the cut-off of 0.75. These lower values may be attributed to variability in the duration of movement phases. In fact, the temporal phase duration showed only moderate to good intra-operator reliability, indicating that time-dependent metrics are more sensitive to variability and potentially less robust. These findings confirm the consistency and robustness of the computed metrics in characterizing UL movement.

Regarding joint kinematics, intra-operator reliability showed excellent for shoulder and wrist kinematics (ICC > 0.906) and good reliability for elbow movements (ICC > 0.755). Inter-operator reliability was good to excellent (shoulder ICC > 0.958, elbow ICC > 0.762, wrist ICC > 0.826) Usability. Slightly lower reliability, ranging from moderate to good, was observed for the thorax and head segments. This is explained by the paravertebral muscles underneath the markers: during movement, their contractions modify the morphology of the back, thus adding an intrinsic 3D displacement to the corresponding markers and producing unavoidable, unremovable artifacts. The reliability values reported in our study are in line with previous findings in UL kinematic protocols [17,18], which report ICC values above 0.75 for most joint-level measures. Overall, these results support the conclusion that the proposed protocol is both reliable and repeatable. In particular, the kinematic data showed at least good repeatability, as well as some spatiotemporal parameters such as MMV, CI and ASI, which quantitatively describe the quality of movement. This set of outcomes, when combined with traditional clinical scales, could serve as a useful tool to support treatment personalization and to evaluate outcomes. Importantly, the protocol is simple to implement and adheres to ISB guidelines, which facilitates its adoption across a range of commercial optoelectronic systems beyond the specific system used in this study.

The proposed protocol provides a simple and efficient framework to calculate the main kinematic and spatiotemporal metrics of UL movement with a limited number of acquisitions. This approach is in line with previous studies on UL movement analysis, which emphasize the importance of balancing measurement accuracy with protocol simplicity and feasibility in clinical settings [17,18]. Furthermore, with respect to these studies, our protocol allows the evaluation of intra—and interoperative repeatability in healthy subjects, demonstrating reliability values higher than good.

The next step will be the clinical application of the protocol: together with clinical data, further validation will be obtained (through an intra and inter-operator reliability analyses) in subjects with UL altered movement patterns like cerebral palsy or acquired brain injury. The proposed protocol is subject to intrinsic limitations such as skin motion artifacts, although previous studies have demonstrated these to be negligible in terms of their impact on reliability [39]. The information related to the UL movement patterns during movement like hit to target is a fundamental outcome of this methodology it could be very useful for clinical diagnosis in several pathologies for an integrated functional assessment. The first one that will be investigated in the next months will be cerebral palsy and acquired brain injury in children.

### Limitations

Despite the encouraging results, this study presents some limitations that should be acknowledged. First, the sample size was relatively small (n = 10), which is adequate for assessing technical reliability and repeatability [26,33], limits the generalizability of the findings to broader or more heterogeneous populations. Future studies including clinical populations and a larger number of participants are necessary to confirm the applicability and robustness of the protocol in real-world clinical scenarios. Lastly, although the protocol was designed to be general-purpose and pediatric-friendly, its performance in specific clinical populations—such as children with neuromotor impairments—has yet to be evaluated. These populations may present additional challenges, such as involuntary movements, limited range of motion, or attention deficits, which could impact marker placement accuracy and task execution.

## 5. Conclusions

This study presents the development and validation of a protocol for the integrated assessment of UL movements, providing a comprehensive analysis of the upper-quarter body kinematics in both pediatric and adult populations. The reliability of the protocol was validated on a sample of 10 healthy subjects, yielding satisfactory and reliable results. Usability assessments from both operators and patients were also positive, supporting the protocol’s practicality in clinical settings. The demonstrated intra- and inter-operator repeatability suggests that this protocol can be confidently introduced into clinical practice for the diagnosis and monitoring of rehabilitative treatments. Furthermore, thanks to its simple implementation following ISB guidelines, the methodology is adaptable to various commercial optoelectronic systems, facilitating its adoption across different clinical environments. Although clinical scales remain the standard for evaluating UL functionality in diagnosis and rehabilitation, this optoelectronic-based protocol provides a reliable, non-invasive method to quantify detailed kinematic parameters relevant for clinical assessment and treatment planning. The collected dataset establishes preliminary normative values, which will be expanded in future studies to develop a comprehensive clinical database for specific pathologies.

## Figures and Tables

**Table 1:** Mean, standard deviation (SD), and range for baseline features of healthy subjects.

Baseline Features	
Age [y] (mean—SD; range)	18.69—SD 12.45; 8.0–41.4
Weight [Kg] (mean—SD; range)	44.5—SD 22.55; 24.00–90.00
Height [cm] (mean—SD; range)	144.5—SD 18.28; 124.0–167.0
BMI (mean—SD; range)	20.03—SD 5.65; 15.06–33.06

**Table 2:** Reference system of the different body segments.

Thorax coordinate system [23]	Origin:	The origin coincided with suprasternal notch (IJ)
	Y axis:	The line connecting the midpoint between xiphoid process (PX) and T8 and the midpoint between IJ and C7, pointing upward
	Z axis:	The line perpendicular to the plane formed by IJ, C7, and the midpoint between PX and T8, pointing to the right.
	X axis:	The common line perpendicular to the Z and Y axis, pointing forwards.
Proximal Humerus (note: This reference frame was used to calculate shoulder movement) [23]	Origin:	The origin coincident with GlenoHomeros (GH)
	Y axis:	The line connecting GH and the midpoint of lateral elbow (EL) and medial elbow (EM)), pointing to GH.
	Z axis:	The common line perpendicular to the Y and Z axis, pointing to the right
	X axis:	The line perpendicular to the plane formed by EL, EM, and GH, pointing forward.
Distal Humerus (note: This reference frame was used to calculate elbow movement) [25]	Origin:	coincides with midpoint of the elbow
	Y axis:	Forearm axis of rotation.
	Z axis:	The line perpendicular to the plane formed by Y and the Y axis of the proximal humerus reference frame
	X axis:	perpendicular to the plane formed by X and Y, POINTing forward
Forearm coordinate system [23]	Origin:	The origin coincided with ulnar styloid (US).
	Y axis:	the line connecting US and the midpoint between EL and EM, pointing proximally
	Z axis:	The common line perpendicular to the X and Y axis, pointing to the right.
	X axis:	The line perpendicular to the plane through US, radial styloid (RS), and the midpoint between EL and EM, pointing forward
Wrist coordinate system [23]	Origin:	The origin of the coordinate systems is located midway between the base and head of second metacarpal. In the transverse plane, it will be at the approximate center of the tubular bone.
	Y axis:	The line parallel to a line from the center of the distal head of the metacarpal to the midpoint of the base of the metacarpal.
	Z axis:	The common line perpendicular to the X and Y axis.
	X axis:	The X and Y axis will form a sagittal plane that splits the metacarpal into mirror images

**Table 3:** Spatiotemporal metrics of movement.

	Metrics	
End-point (finger)	Time	This is calculated as the total time required for completing each task. In addition, the durations of the previous three phases were computed: the going phase, the adjusting phase, and the returning phase [31]
	Adjusting sway Index (ASI)	It is defined as the length of the 3D path described by the fingernail during the adjusting phase, which is a measure of the adjustments made to reach the final position. This represents an expression of the degree of precision [31].
	Mean Movement Velocity (MMV)	It is computed during the going phase and represents the mean velocity of the fingernail marker [31].
	Index of curvature (IC)	It is calculated as the ratio of the fingernail 3D path length to the linear distance between the initial and the final pointing position and is a representative of movement smoothness during the going phase [32].
	Number of Velocity Peaks (NVP)	It is a quality measure of movement smoothness computed from the speed profile of the finger during the entire movement [32].
Trunk compensation	Trunk compensation	It is defined as the length 3D path described by the marker placed on the sternum [33]
	Displacement along z-axis	Displacement of the marker placed on the sternum along the z-axis (towards the target) during the going phase. It quantifies trunk flexion [33].
	Displacement along x-axis	Displacement of the marker placed on the sternum along the x-axis during the going phase. It quantifies trunk lateral bending [33].

**Table 4:** Intraclass correlation coefficient (ICC) and their levels about end-point metrics for intra and inter operator analysis (+++ = excellent, ++ = good, + = moderate, − poor) [37].

	Intra Operator		Inter Operator	
	ICC	ICC Level	ICC	ICC Level
T tot	0.785	++	0.715	+
T Going Phase	0.738	+	0.610	+
T Return Phase	0.725	+	0.552	+
T Adj Phase	0.839	++	0.816	++
Mean movement velocity (MMV)	0.896	++	0.953	+++
Curvature Index (CI)	0.898	++	0.898	++
Adjusting sway Index (ASI)	0.867	++	0.793	++
Number of Velocity Peaks (NVP)	0.739	+	0.721	+

**Table 5:** Intraclass correlation coefficient (ICC) and their levels about trunk indexes for inter-operator analysis (+++ = excellent, ++ = good, + = moderate, − poor) [37].

	Intra Operator		Inter Operator	
	ICC	ICC Level	ICC	ICC Level
Thorax Length Going Phase	0.815	++	0.834	++
Antero-posterior Going Phase Displacement	0.818	++	0.890	++
Medio-lateral Going Phase Displacement	0.768	++	0.696	+
Thorax Length Adjusting Phase	0.926	+++	0.834	++
Antero-posterior Adjusting Phase Displacement	0.882	++	0.707	+
Medio-lateral Adjusting Phase Displacement	0.956	+++	0.854	++
Thorax Length Return Phase	0.870	++	0.870	++
Antero-posterior Return Phase Displacement	0.891	++	0.888	++
Medio-lateral Return Phase Displacement	0.815	++	0.825	++

**Table 6:** Intraclass correlation coefficient (ICC) and their levels about range of motion (ROM) for intra and inter operator analysis (+++ = excellent, ++ = good, + = moderate, − poor) [37].

	Intra Operator		Inter Operator	
	ICC	ICC Level	ICC	ICC Level
ROM Right Shoulder FE	0.983	+++	0.992	+++
ROM Left Shoulder FE	0.991	+++	0.992	+++
ROM Right Shoulder AA	0.906	+++	0.992	+++
ROM Left Shoulder AA	0.966	+++	0.958	+++
ROM Right Shoulder IE	0.917	+++	0.965	+++
ROM Left Shoulder IE	0.949	+++	0.954	+++
ROM Right Elbow FE	0.959	+++	0.950	+++
ROM Left Elbow FE	0.978	+++	0.953	+++
ROM Right Elbow PS	0.755	++	0.848	++
ROM Left Elbow PS	0.838	++	0.762	++
ROM Right Wrist FE	0.967	+++	0.826	++
ROM Left Wrist FE	0.949	+++	0.941	+++
ROM Right Wrist UR	0.934	+++	0.952	+++
ROM Left Wrist UR	0.949	+++	0.940	+++
ROM Right Thorax FE	0.869	++	0.952	+++
ROM Right Thorax ML	0.640	+	0.872	++
ROM Right Thorax IE	0.768	++	0.722	+
ROM Right Head FE	0.705	+	0.867	++
ROM Right Head ML	0.813	++	0.857	++
ROM Right Head IE	0.832	++	0.798	++

**Table 7:** Assessment of the difficulty of the application of the protocol (1: very difficult–5: very easy).

	Operators				Patient		
Phase	1	2	3	4	1	2	3
Mean	4.83	4.68	4.95	4.98	4.88	4.90	4.70
Standard deviation	0.38	0.47	0.22	0.16	0.33	0.30	0.56
Max	5.00	5.00	5.00	5.00	5.00	5.00	5.00
Min	4.00	4.00	4.00	4.00	4.00	4.00	3.00

**Table 8:** Time required for the entire acquisition and for each phase. The phase1 correspond to the instrument preparation, phase 2 correspond to the marker positioning, phase 3 correspond to recording the movements with the optoelectronic system and the phase 4 correspond to the removing the markers.

T [min]					
	1	2	3	4	TOT
Mean	2.00	6.05	10.13	1.80	19.97
Standar deviation	0.16	0.62	0.54	0.16	0.69
Max	2.32	7.22	11.73	2.10	21.50
Min	1.77	5.17	8.85	1.20	18.73

## Data Availability

Data presented in this study are available on request to the corresponding author in anonymous form due to GDPR and informed consent.

## Abbreviations

The following abbreviations are used in this manuscript:

UL	Upper Limb
CP	Cerebral Palsy
MUUL	The Melbourne Assessment of Unilateral Upper Limb Function
QUEST	Quality of Upper Extremity Skills Test
SHUEE	Shriners Hospital Upper Extremity Evaluation
CIMT	Constraint-Induced Movement Therapy
TRT	Trunk Restraint Therapy
BAT	Bilateral Arm Therapy
ICF	International Classification of Functioning
ISB	International Society of Biomechanics
HtT	Hit-to-Target
SUS	System Usability Scale
ROM	Range of motion
ICC	intraclass correlation coefficient
MMV	Mean Movement Velocity
IC	Index of curvature
NVP	Number of Velocity Peaks
MA	motion analysis
